# Child Posttraumatic Stress after Parental Cancer: Associations with Individual and Family Factors

**DOI:** 10.1093/jpepsy/jsac041

**Published:** 2022-05-20

**Authors:** Marthe R Egberts, Dineke Verkaik, Anneloes L van Baar, Trudy T M Mooren, Mariken Spuij, Liesbeth G E de Paauw-Telman, Paul A Boelen

**Affiliations:** Department of Clinical Psychology, Utrecht University, The Netherlands; Ingeborg Douwes Centrum, Centre for Psycho-oncology, The Netherlands; Department of Clinical Psychology, Utrecht University, The Netherlands; Child and Adolescent Studies, Utrecht University, The Netherlands; Child and Adolescent Studies, Utrecht University, The Netherlands; Department of Clinical Psychology, Utrecht University, The Netherlands; ARQ National Psychotrauma Centre, The Netherlands; Child and Adolescent Studies, Utrecht University, The Netherlands; TOPP-zorg, The Netherlands; Child and Adolescent Studies, Utrecht University, The Netherlands; Department of Clinical Psychology, Utrecht University, The Netherlands; ARQ National Psychotrauma Centre, The Netherlands

**Keywords:** oncology, parent–adolescent communication, parent psychosocial functioning, parental illness, posttraumatic stress and trauma, psychosocial functioning

## Abstract

**Objective:**

This study aimed to examine the severity of posttraumatic stress disorder (PTSD) symptoms in children of parents with cancer and to identify individual and family factors associated with these symptoms.

**Methods:**

The sample consisted of 175 children (52% girls, aged *M *=* *11.98, SD* *=* *3.20, range = 6–20 years) from 92 families, of which 90 parents with a current or past cancer diagnosis and 71 healthy co-parents also completed questionnaires. Children reported on PTSD symptoms, trauma-related cognitions, emotion regulation difficulties, general family functioning, and family communication. Both parents reported on their own PTSD symptoms. Associations were investigated using multilevel regression.

**Results:**

Twenty-seven percentage of the children showed clinically relevant PTSD symptoms. Intraclass correlations indicated that children from the same family showed little overlap in these symptoms. Multilevel analyses showed that child trauma-related cognitions and emotion regulation difficulties were related to higher levels of PTSD symptoms at the individual level. General family functioning was only related to child PTSD symptoms at the family level. Child PTSD severity was unrelated to parental PTSD symptoms and family communication at the family level when taking into account the other factors.

**Conclusions:**

The current study highlights the psychological impact of parental cancer on children. Individual factors contributed more strongly to child PTSD symptoms than family factors. Trauma-related cognitions and emotion regulation difficulties might be targeted through specific psychoeducation for children and parents, family-oriented support and interventions, and evidence-based treatments for child PTSD.

## Introduction

Parental cancer is a stressor for children that can elicit symptoms of posttraumatic stress disorder (PTSD). The life threat associated with the diagnosis, witnessing the parent going through intensive treatment, insecurities regarding prognosis, and changes at home can be very stressful for children. Clinically relevant child PTSD symptoms have been reported for 21% of sons and 35% of daughters (11–23 years old) 1–5 years after their parent’s diagnosis (Huizinga et al., 2005b). In a previous paper on the same sample as used in the current study, four different patterns of child adjustment were identified, based on children’s health-related quality of life (HRQoL), PTSD symptoms, and satisfaction with life (Egberts et al., 2021). Whereas the majority of children (75%) displayed average to high levels of functioning, 25% of children experienced high levels of posttraumatic stress and impaired HRQoL, in some cases combined with low life satisfaction. PTSD symptoms in children may have adverse consequences for their emotional and cognitive development (Davis & Siegel, 2000). The current study was designed to provide more insight into risk and protective factors for PTSD symptoms in children of parents with cancer, through which targets for intervention might be identified.

The wider literature on PTSD emphasizes the role of negative trauma-related cognitions or appraisals in relation to an individual’s adjustment to stressful events (Ehlers & Clark, 2000; Foa et al., 1999). It has been suggested that negative appraisals of trauma and its consequences lead to a sense of threat that maintains PTSD symptoms (Ehlers & Clark, 2000). Empirically, negative trauma-related cognitions about the self, others, and the world have been related to higher levels of PTSD symptoms concurrently and longitudinally (Ehring et al., 2008; O’Donnell et al., 2007). These associations have also been shown in children (De Haan et al., 2019; Meiser-Stedman et al., 2009a; Mitchell et al., 2017). However, child trauma-related cognitions have not been examined in the context of parental cancer as such. The examination of cognitions has mainly been limited to cognitive appraisals of the seriousness of the parent’s illness (Huizinga et al., 2005b; Nelson & While, 2002). Nevertheless, qualitative research indicated that a wider array of trauma-related cognitions may be present in children confronted with parental cancer, for example relating to the emotional impact of the parent’s disease, the threat of parental death, and worries about the future (Kennedy & Lloyd-Williams, 2009; Marshall et al., 2021).

Emotion regulation is another factor that has received considerable attention in relation to PTSD symptoms (Chesney & Gordon, 2017; McLean & Foa, 2017). Emotion regulation refers to managing one’s internal experience and external expression of emotions (Gross, 1998). A meta-analysis suggested a strong cross-sectional association between emotion regulation difficulties and PTSD symptoms in children and adolescents across various trauma types (Villalta et al., 2018). Similar to trauma-related cognitions, child emotion regulation difficulties have received little attention in the context of parental cancer. A few studies examined the role of specific coping strategies, including emotion-focused coping, in relation to child adjustment to parental cancer (Compas et al., 1996; Krattenmacher et al., 2013; Thastum et al., 2008). One of these studies showed that some emotion-focused strategies, such as acceptance and social support seeking, were related to better functioning, whereas avoidance-oriented strategies, such as distraction and wishful thinking, were related to worse adjustment in children (Krattenmacher et al., 2013). However, little is known about the broader construct of emotion regulation (i.e., including emotional awareness, clarity of emotional experiences, and managing emotions) and about the unique contribution of emotion regulation and trauma-related cognitions to child PTSD symptoms.

Parents with cancer and their partners may also experience PTSD symptoms (Huizinga et al., 2011). Parent psychological problems are one of the most consistent predictors of child psychological adjustment to parental cancer (Krattenmacher et al., 2012). For instance, depressive symptoms in parents with cancer have been related to more child internalizing symptoms (Thastum et al., 2009). In addition, one study reported a positive association between parent and child PTSD symptoms (Huizinga et al., 2011). However, that study did not differentiate between posttraumatic stress of the parent with cancer and the healthy partner. Within families, the healthy parent may play a compensating role for the parent with cancer when necessary, which may be a protective factor in the child’s adjustment (Lewis & Darby, 2003; Visser et al., 2006). It is, therefore, relevant to examine the reactions of both the ill and healthy parent to better understand the adjustment of the family system.

Alongside individual factors, family factors also affect children’s adjustment to parental cancer. Better family functioning appears to be associated with better child adjustment (i.e., lower levels of emotional and behavioral problems) in families confronted with cancer (Krattenmacher et al., 2012; Lindqvist et al., 2007; Thastum et al., 2009; Watson et al., 2006). A more specific family factor is family communication. Although the wider literature on parental cancer emphasizes the importance of open communication between parents and children about the illness (Ellis et al., 2017; Su & Ryan-Wenger, 2007), only a few studies have empirically studied family communication in relation to child adjustment (Huizinga et al., 2005a, 2011; Lindqvist et al., 2007; Nelson & While, 2002). Most of these studies show that open family communication is related to lower levels of emotional and behavioral problems, including PTSD symptoms (Huizinga et al., 2005a, 2011; Lindqvist et al., 2007), albeit that at least one study did not find this relationship (Nelson & While, 2002) and that one of the studies found different relationships for boys and girls (Huizinga et al, 2005a). In addition, knowledge gaps remain regarding the interplay between these family factors and individual child factors.

Taken together, previous studies suggest that certain child cognitive and emotional factors as well as parents’ PTSD symptoms might place children at risk of developing symptoms of PTSD, whereas better family functioning and communication may play a more protective role. However, these factors have not been examined in one single analysis yet, so conclusions about their relative impact on child’s functioning cannot be drawn. In addition, to our knowledge, only three previous studies (Huizinga et al., 2011; Krattenmacher et al., 2013; Möller et al., 2014) have taken into account the dependent nature of the family data in their statistical analyses (i.e., children and parents belong to the same family and therefore share certain characteristics). In the current study, multilevel analyses were used to handle the dependency of the data and to differentiate between individual and family factors.

The aim of the current study was to examine the level of PTSD symptoms in children of parents with cancer and to identify individual and family factors associated with these symptoms. It was hypothesized that both individual and family factors would make a unique contribution to child PTSD symptoms (Huizinga et al., 2011). On the individual level, more child trauma-related negative cognitions (Mitchell et al., 2017) and emotion regulation difficulties (Krattenmacher et al., 2013; Villalta et al., 2018) were expected to show a positive relationship with PTSD symptoms. No significant associations for child age and gender were anticipated (Krattenmacher et al., 2012). At the family level, the severity of parental PTSD symptoms was expected to be associated with the severity of PTSD symptoms in children (Huizinga et al., 2011). In addition, better family functioning and family communication were hypothesized to relate to lower levels of child PTSD symptoms (Huizinga et al., 2005a; Thastum et al., 2009). No associations between child’s PTSD symptoms and illness phase were expected (Krattenmacher et al., 2012).

## Methods

### Participant Recruitment and Procedure

This study is part of a larger longitudinal study on family adjustment in the context of parental cancer. The current study relies on data collected at the first timepoint. A previous article on this study reported on latent classes of child adjustment, based on profiles of PTSD symptoms, HRQoL, and satisfaction with life (Egberts et al., 2021). Families were eligible to participate if one of the parents had a current or past cancer diagnosis and had at least one child in the age of 0–18 years. Exclusion criteria included limited Dutch language proficiency and terminal stage of cancer. Between September 2015 and April 2019, and between November 2019 and February 2020 (after a change in personnel involved in this project), families were recruited through social media announcements, personal contact with health care providers, and support centers for cancer patients. Families received information about the study to consider participation. After signing up, parents were contacted by telephone to receive additional study information and share further information through a short semi-structured interview, containing questions about the type and stage of cancer, illness and treatment phase, and family characteristics (e.g., marital state and major life events in the family). Since recruitment was not carried out from hospitals, the focus of the interview was on family psychosocial adjustment, rather than medical information. After the interview, around 25 parents decided not to participate because they regarded participation too demanding for themselves or their children. Data were obtained through questionnaires filled out by parents and children. Children from 8 to 18 years old^1^ were eligible to participate themselves. Home visits were carried out to gather the questionnaire data, primarily focused on supporting (younger) children in filling out the questionnaires. Most home visits were carried out by trained master-students. Alternatively, families could choose to fill out the questionnaires themselves and return them by post. Family members were instructed to complete questionnaires independently and to not discuss their answers amongst themselves. Written informed consent was obtained from participating parents and children. The study was approved by the local ethics committee of the Faculty of Social and Behavioral Sciences of Utrecht University (FETC15-061). The data underlying this article cannot be shared publicly due to the privacy of the participants. The data will be shared on reasonable request to the corresponding author.

### Participants

In total, 136 families were enrolled in the project. For the purpose of the present study, families were included in the analyses when at least one child completed the child PTSD symptoms assessment (i.e., the study’s principle dependent variable). Parent data were included if the parent completed the parent PTSD symptoms measure. This resulted in a sample of 175 children from 92 different families, of which 90 parents with cancer and 71 healthy parents completed the measures. In two participating families, the parent with cancer was hospitalized and not in the condition to complete the questionnaires. The sample characteristics, including illness characteristics of 92 parents, are displayed in Table I. Children had a mean age of 11.98 (SD* *=* *3.20, range = 6–20^1^) years. In the majority of the families, the mother was diagnosed with cancer (85%). In none of the families, both parents were diagnosed with cancer. The mean time since diagnosis was 2.85 years (SD* *=* *2.89). Most participating parents were of Dutch origin (99%).

**Table I.** Sample Characteristics

Child characteristics (n = 175)		
Gender [*n* (%)]		
Girl	92 (52)	
Boy	82 (47)	
Non-binary	1 (1)	
Age (in years) [*M (SD)*]	11.98 (3.20)	
Current education [*n* (%)]		
Primary school	91 (52)	
Secondary school	76 (43)	
Other	8 (5)	
Participating parents’ characteristics	Parent with cancer (*n* = 90)	Healthy parent (*n* = 71)
Gender [*n* (%)]		
Female	78 (87)	14 (20)
Male	12 (13)	57 (80)
Age (in years) [*M (SD)*]	44.06 (6.11)	44.96 (6.46)
Highest education [*n* (%)]		
Primary/secondary school	33 (37)	35 (49)
College/university	57 (63)	36 (51)
Relation to child		
Biological parent	90 (100)	67 (94)
Stepparent	0 (0)	4 (6)
Country of birth [*n* (%)]		
The Netherlands	89 (99)	70 (99)
Morocco	1 (1)	1 (1)
Illness characteristics (of 92 parents with cancer)		
Cancer type [*n* (%)]		
Breast cancer	66 (72)	
Gastrointestinal cancer	8 (9)	
Hematological cancer	5 (5)	
Testicular cancer	3 (3)	
Other malignancies	10 (11)	
Metastasized cancer [*n* (%)]		
Yes	33 (62)	
No	57 (36)	
No information available	2 (2)	
Illness phase [*n* (%)]		
Active treatment (e.g., chemotherapy or radiation therapy) or not started treatment yet	37 (40)	
First year after treatment (with possibility of receiving hormone therapy)	19 (21)	
1–5 years after treatment	21 (23)	
5–10 years after treatment	9 (10)	
Palliative	5 (5)	
No information available	1 (1)	

### Measures

#### Child Posttraumatic Stress Symptoms

Child-reported PTSD symptoms were assessed with the Child PTSD Symptoms Scale (CPSS; Foa et al., 2001). Children rated their symptoms in relation to the parent’s cancer and its consequences. The CPSS assesses the frequency of 17 PTSD symptoms (as per DSM-IV-TR; American Psychiatric Association, 2000) throughout three symptom clusters: re-experiencing, avoidance, and hyperarousal. The 17 symptom-items are rated on a 4-point Likert scale (0 = *not at all*, 1 = *once a week or less*, 2 = *2–4 times a week*, 3 = *5 or more times per week*). The total score is calculated by summing all item scores. A total score of 16 or higher was used to indicate clinical relevance of symptoms (i.e., the presence of probable PTSD) (Nixon et al., 2013). Cronbach’s alpha of the total scale was 0.85 in the current study.

#### Child Trauma-Related Cognitions

The Dutch version (Diehle et al., 2015a) of the Child Posttraumatic Cognitions Inventory (cPTCI; Meiser-Stedman et al., 2009b) was used to assess child self-reported trauma-related cognitions. This questionnaire consists of 25 items rated on a 4-point Likert scale (from 1 = *don’t agree at all* to 4 = *agree a lot*). The total score is calculated by summing all item scores. Subscale scores are calculated for the subscales “permanent and disturbing change” and “fragile person in a scary world”. Cronbach’s alpha of the total scale, included in the analyses for this study, was 0.91.

#### Child Emotion Regulation Difficulties

The Difficulties in Emotion Regulation Scale (DERS; Gratz & Roemer, 2004) was administered to assess child difficulties in emotion regulation. The DERS consists of 36 items rated on a 5-point Likert scale (ranging from 1 = *almost never* to 5 = *almost always*). The scale is divided into six subscales: lack of emotional awareness (six items), lack of emotional clarity (five items), difficulties controlling impulsive behaviors when distressed (six items), difficulties engaging in goal-directed behavior when distressed (five items), non-acceptance of negative emotional responses (six items), and limited access to effective emotion regulation strategies (eight items). A total DERS score is calculated by summing all item scores. Cronbach’s alpha of the overall scale was 0.90.

#### General Family Functioning and Family Communication

The McMaster Family Assessment Device (FAD; Epstein et al., 1983) was administered to measure child-reported family functioning. Children filled out the 60-item questionnaire. The FAD is composed of seven subscales, of which two were used for the purpose of the current study, namely “general functioning” (12 items assessing the overall health/pathology of the family) and “communication” (nine items assessing the exchange of information among family members, e.g., “we are frank with each other”). Questions are answered on a 4-point Likert scale (ranging from 1 =* strongly agree* to 4 = *strongly disagree*). Subscale mean scores were calculated, with higher scores indicating better functioning. The instrument can be used for children 12 years and older (Epstein et al., 2000). In the current study, children under 12 years also completed the FAD. When needed, the master-students who carried out the home visits provided (standardized) clarification to the items. The reliability of the “general functioning” subscale was good (Cronbach’s alpha = 0.81), whereas the reliability of the “communication” subscale was minimally acceptable (Cronbach’s alpha = 0.64).

#### Parent Posttraumatic Stress Symptoms

Both parents completed the PTSD Symptom Scale-Self Report (PSS-SR; Foa et al., 1993). The PSS-SR assesses 17 PTSD symptoms, divided into three symptom clusters (according to DSM-IV). Items are scored on a 4-point Likert scale (0 = *not at all*, 1 = *once a week or less*, 2 = *2–4 times a week*, 3 = *5 or more times per week*). The total PSS-SR scale demonstrated good reliability for the parent with cancer (Cronbach’s alpha = 0.91) and the healthy parent (Cronbach’s alpha = 0.93).

#### Socio-demographic and Illness Characteristics

Parents and children completed a questionnaire on socio-demographic characteristics. Parents’ self-reported on their nationality and country of birth through an open question. They also indicated which parent was diagnosed with cancer. Information regarding illness phase was obtained during the semi-structured interview conducted by telephone. Illness phase was divided into the following categories: no treatment yet, active treatment (e.g., chemotherapy or radiation therapy), first year after treatment (with possibility of receiving hormone therapy), 1–5 years after treatment, 5–10 years after treatment, cancer in palliative phase. No parents with cancer in the terminal phase were included.

### Statistical Analyses

First, descriptive statistics for child and parent PTSD symptoms and child-reported trauma-related cognitions (total cPTCI score), emotion regulation difficulties (total DERS score), general family functioning, and family communication were calculated.

Multilevel regression analyses with random intercepts were used to examine individual and family predictors of child PTSD symptoms. These analyses were used because of nested data (i.e., children are nested within families) and allowed to account for the non-independence of observations. The data had a two-level hierarchy, with the individual child on the lowest level (first level) and the family on the highest level (second level). The variance of the outcome variable (child PTSD symptoms) was separated across the two levels. Predictors on the first level included child gender, age, trauma-related cognitions, and emotion regulation difficulties. Predictors on the second level entailed the parent’s illness phase and PTSD symptoms of both the parent with cancer and the healthy parent. Child-reported general family functioning and family communication were examined at the first level (i.e., comprising the child’s individual perception of the family’s functioning) as well as the second level. The variables at the second level were aggregated variables constructed from the first level variables, comprising the *mean level* of child-reported general family functioning and family communication of children from the same family. Information regarding illness phase was collapsed into two categories, based on previous research (Rolland, 2005). The first category concerned the crisis phase (i.e., parents who received active treatment or had not started treatment yet); the second category comprised the chronic or adaptation phase (i.e., parents within the first year up to 10 years after treatment or parents with cancer in the palliative phase). Analyses were conducted in Mplus 7.4 (Muthén & Muthén, 2010). A robust maximum likelihood estimator was used because some variables were non-normally distributed.

Full information maximum likelihood (FIML) was used to estimate missing data, allowing all available information to be used. This estimation requires a missing conditionally at random assumption (Hox et al., 2018). The reasonableness of this assumption was evaluated through missing data analyses.

**Figure 1.**
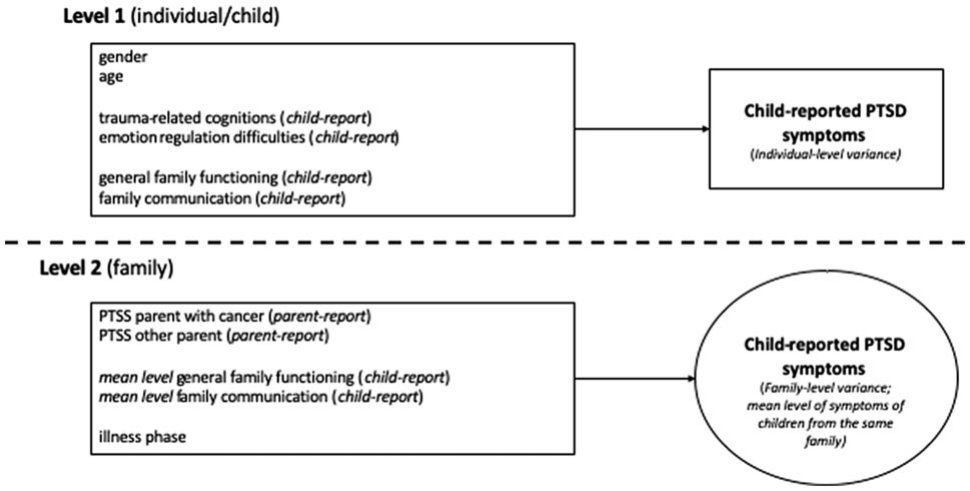
Final multilevel model including all modeled predictors of child PTSD symptoms on the individual child and family level.

In the first step of the multilevel analyses, a baseline model was estimated, which included the variance of child PTSD symptoms and the continuous predictor variables. Intraclass correlations were calculated to define the proportion of variance of child PTSD symptoms at each level. Before estimating the final model, univariate multilevel correlations between predictor and outcome variables were calculated, including random intercepts. In the next step, the regression paths of the predictor variables on the individual child level were added to the model, followed by the variables on the family level (see Figure 1 for an overview of all variables included in the final model). All continuous predictor variables were grand-mean centered. The final model including the family predictors was compared to the baseline model to examine improvement in model fit, using a chi-square difference test based on the models’ loglikelihood values.

## Results

### Descriptive Statistics

Table II displays the means and standard deviations of the study variables; 27% of the children reported clinically relevant symptoms of PTSD (i.e., total score of ≥16 on the CPSS). Child PTSD levels did not differ for children from parents with metastasized cancer, compared to children from parents with non-metastasized cancer (*p* = .56). The mean levels of child-reported family functioning and communication were relatively high.

**Table II.** Univariate Multilevel Correlations, Means (M), and Standard Deviations (SD) of the Study Variables on Two Levels

	1.	2.	3.	4.	5.	*M*	*SD*	Range	*N*
Individual child level (1)									
1. Child PTSD symptoms	–					11.00	8.17	0.00–42.00	175
2. Trauma-related cognitions	0.61**	–				37.36	10.81	25.00–78.00	174
3. Emotion regulation difficulties	0.59**	0.57**	–			87.43	21.47	46.00–147.00	173
4. Child-reported general family functioning	–0.16*	–0.20**	–0.25**	–		3.23	0.44	1.75–4.00	173
5. Child-reported family communication	–0.23**	–0.30**	–0.38**	0.67**	–	3.01	0.39	2.11–3.89	173
6. Age	0.01	0.12	0.12	–0.04	–0.08	11.98	3.20	6.00–20.00	175
Family level (2)									
1. Child PTSD symptoms	–								
2. PTSD symptoms parent with cancer	0.19	–				15.38	9.09	0.00–41.00	90
3. PTSD symptoms healthy parent	–0.05	0.22	–			13.00	10.12	0.00–46.00	71

*Note.* Multilevel correlations are based on *n *=* *172 children from 91 families. PTSD = Posttraumatic Stress Disorder.

* *p* < .05; **p < .01.

### Missing Data Analyses

Data of children were included in the final multilevel model if they completed the CPSS and had complete information on the dichotomous predictors included in the model (i.e., child gender and parent illness phase). Incomplete data on continuous predictors were allowed, because these could still be included with the use of FIML (Hox et al., 2018). This selection resulted in a sample of 172 children from 91 families, including data from 89 parents with cancer and 71 healthy parents. For 77% of the children, data on continuous predictor variables were complete. For 21% of the children, data on one of the continuous predictor variables was missing. For 2%, this was the case for two variables or more. Children with complete data did not differ from children with missing data in terms of age (*p* = .21), gender (*p* = .11), or child PTSD symptoms (*p* = .76).

### Multilevel Analyses

#### Unexplained Variance

Intraclass correlations showed that 88% of the variance in child PTSD symptoms was located at the individual child level (level 1) and 12% at the family level (level 2). This indicates that PTSD symptoms of children from the same family showed little overlap. Nevertheless, to correct for the nested structure of the data, we modeled random intercepts in multilevel analyses.

#### Univariate Correlations

Correlations between the different predictor variables, and between the predictor and outcome variables, were calculated for the two levels separately using random intercepts (see Table II). On the individual child level, significant univariate associations were found: more PTSD symptoms were related to higher levels of trauma-related cognitions and emotion regulation difficulties, and worse child-reported family functioning and communication. The predictor variables were also significantly related: higher levels of trauma-related cognitions and emotion regulation difficulties were associated with worse child-reported family functioning and communication. On the family level, no significant univariate associations were found.

#### Multivariate Associations

**Figure 2.**
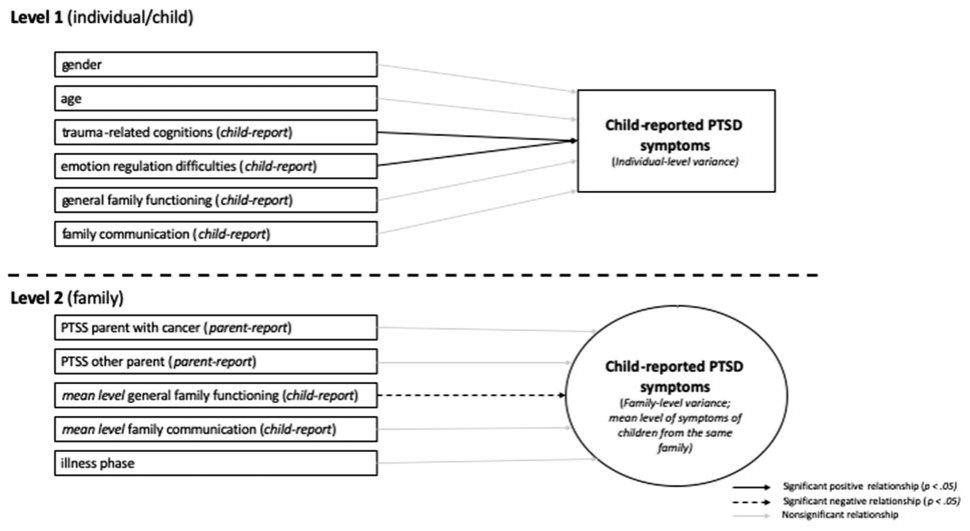
Results of final multilevel model (Model 3) including all modeled predictors of child PTSD symptoms on the individual child and family level.

Results of the multilevel regression analyses are presented in Table III. A summary of the main findings is schematically depicted in Figure 2. In the final model (Model 3), at the individual child level, more child trauma-related cognitions (*B *=* *0.28, *p* < .001) and emotion regulation difficulties (*B* = 0.15, *p* < .001) were related to higher levels of PTSD symptoms, as hypothesized. At the individual child level, no associations were found for child-reported family functioning (*B *=* *1.40, *p* = .55) and communication (*B *=* *–1.29, *p* = .58), which was unexpected. As hypothesized, no associations were found for child gender (*B *=* *1.70, *p* = .07) and age (*B *=* *–0.21, *p* = .16).

**Table III.** Multilevel Regression Analyses: Child and Family Predictors of Child PTSD Symptoms

	Model 1		Model 2		Model 3	
	*B* (SE)	95% CI	*B* (SE)	95% CI	*B* (SE)	95% CI
Individual child level—child PTSD symptoms						
Gender (male = 0, female = 1)	1.77 (0.92)	–0.04 to 3.58	1.70 (0.93)	–0.13 to 3.53	1.70 (0.93)	–0.12 to 3.52
Age	–0.24 (0.15)	–0.53 to 0.05	–0.20 (0.15)	–0.49 to 0.09	–0.21 (0.15)	–0.51 to 0.08
Trauma-related cognitions	0.30** (0.04)	0.22 to 0.38	0.28** (0.04)	0.20 to 0.37	0.28** (0.04)	0.20 to 0.36
Emotion regulation difficulties	0.14** (0.03)	0.08 to 0.20	0.15** (0.03)	0.10 to 0.20	0.15** (0.03)	0.10 to 0.20
Child-reported general family functioning	0.08 (1.93)	–3.71 to 3.86	0.24 (1.89)	–3.48 to 3.95	1.40 (2.32)	–3.15 to 5.95
Child-reported family communication	0.27 (2.15)	–3.96 to 4.48	–0.07 (2.10)	–4.19 to 4.04	–1.29 (2.32)	–5.83 to 3.25
Family level—mean level of child PTSD symptoms						
Illness phase (0 = crisis, 1 = chronic/adaptation)			–1.68 (0.91)	–3.45 to 0.10	–1.65 (0.90)	–3.41 to 0.11
PTSD symptoms parent with cancer			0.03 (0.06)	–0.09 to 0.14	0.03 (0.06)	–0.09 to 0.14
PTSD symptoms healthy parent			–0.02 (0.06)	–0.14 to 0.10	–0.01 (.06)	–.13 to .11
Child-reported general family functioning^a^					–10.71* (4.99)	–20.49 to –.93
Child-reported family communication^a^					7.75 (5.48)	–2.99 to 18.49
Explained variance						
At individual child level	40% (of initial 88%)		40% (of initial 88%)		42% (of initial 88%)	
At family level	–		20% (of initial 12%)		77% (of initial 12%)	

*Note.* Total *N *=* *172 children from 91 families. SE = standard error; CI = confidence interval; PTSD = posttraumatic stress disorder.

a Aggregated variables.

* *p* < .05; **p < .01.

For child PTSD symptoms at the family level (i.e., the mean level of PTSD symptoms for children from the same family), contrary to the hypothesis, no significant associations were found for parent PTSD symptoms (*B*_parent with cancer_ = 0.03, *p* = .64; *B*_healthy parent_ = –0.01, *p* = .84). As hypothesized, the aggregated variable of child-reported general family functioning was significantly associated with child PTSD symptoms such that lower family functioning was related to more PTSD symptoms (*B *=* *–10.71, *p* = .03) and no significant relation was found for illness phase (*B *=* *–1.65, *p* = .07). At the family level, the aggregated variable of family communication was unrelated to child PTSD severity (*B *=* *7.75, *p* = .16), which contrasted the hypothesis. The chi-square difference test indicated that the final model had a significantly better model fit compared to the baseline model (*p* < .001).

## Discussion

The current study is one of the first studies that simultaneously examined the role of individual and family factors in relation to the severity of child PTSD symptoms in families confronted with parental cancer. A first main finding was that 27% of the children experienced clinically relevant PTSD symptoms; this result is generally consistent with prior work showing that although the larger majority of children confronted with parental cancer does not experience pervasive traumatic stress, a significant minority does (Huizinga et al., 2005b). A second main finding was that symptoms of children within the same family showed little overlap, as evidenced by the intraclass correlations. Finally, a third main finding was that individual factors, namely child trauma-related cognitions and emotion regulation difficulties, had a relatively higher contribution to child posttraumatic stress compared to the family factors that we examined. Parent PTSD symptoms were not found to play a significant role, whereas general family functioning was only related to child symptoms on the family level. As hypothesized, child age, gender, and the illness phase of the parent were unrelated to child PTSD symptoms.

Higher levels of trauma-related cognitions and emotion regulation difficulties were related to more severe PTSD symptoms in children, as anticipated. This corresponds with the cognitive model of PTSD (Ehlers & Clark, 2000) and with studies that showed the contribution of these factors in explaining posttraumatic stress in children confronted with other types of trauma (De Haan et al., 2019; Meiser-Stedman et al., 2009a; Mitchell et al., 2017). Qualitative research indicated that children of parents with cancer may experience trauma-related cognitions about the negative effects of parental cancer and its implications for the future, as well as negative views of themselves, others, and the world (Kennedy & Lloyd-Williams, 2009; Marshall et al., 2021). By systematically assessing trauma-related cognitions, the current findings corroborate this prior work, indicating that these cognitions comprise a risk factor for child adjustment. Simultaneously, child emotion regulation difficulties were related to PTSD symptoms. A lack of emotional awareness and difficulties in accepting or managing negative emotions may limit the child’s capacity to cope with a severe stressor such as parental cancer. This extends previous research that focused on the role of specific emotion regulation strategies, such as social support seeking, distraction, and wishful thinking (Krattenmacher et al., 2013), by showing that a broader range of child emotion regulation difficulties is associated with the child’s adjustment. Because of the study’s cross-sectional nature, it remains unknown whether emotion regulation difficulties were present before or developed after the parent was diagnosed with cancer, and whether they acted as a predisposing factor for PTSD symptoms or were a consequence of these symptoms (Villalta et al., 2018). Longitudinal research is needed to shed further light on this.

On the family level, PTSD symptoms of the parent with cancer and the partner were not related to child symptoms of PTSD. This is in contrast with other studies showing that parent mental health problems were negatively associated with child adjustment in the context of parental cancer (Krattenmacher et al., 2012). A possible explanation for these differences is that most prior studies focused on parents’ depressive symptoms and child emotional and behavioral problems, rather than parent and child posttraumatic stress (Thastum et al., 2009; Watson et al., 2006). Parents’ depressive symptoms may be more noticeable to children than parents’ PTSD symptoms and therefore possibly more strongly related to the child’s functioning, although this explanation remains speculative. Also, comparability is limited by the fact that most studies have not corrected for the nested data structure (Krattenmacher et al., 2012). To our knowledge, only one multilevel study has examined the parent–child association in posttraumatic stress in this population, and found this association to be positive (Huizinga et al., 2011). However, that study examined a combined score of PTSD symptoms of parents with cancer and their partners. The differing findings in studies done so far call for more studies examining the co-occurrence of self-reported PTSD symptoms in children, parents with cancer, and their partners.

Univariate correlations indicated that worse child-perceived family functioning and communication were related to more child PTSD symptoms. However, in the multivariate model accounting for the role of trauma-related cognitions and emotion regulation difficulties, no significant associations were found for family functioning and communication at the individual child level. This may be interpreted in at least two ways. First, the child’s individual manner of appraising and coping with their parent’s cancer may be more important for its adjustment than the way in which the family’s functioning is perceived. Second, there may be a more complex interplay between the child’s cognitions, emotion regulation, and family functioning than modeled in the current study. For example, better family functioning may foster the development of emotion regulation skills which, in turn, may decrease the risk of PTSD symptoms. This potential interplay is reflected in the significant univariate associations between the predictors (e.g., emotion regulation difficulties were related to worse family functioning) and requires further investigation using longitudinal designs.

Although the overlap of child PTSD symptoms within the family was small, we examined predictors of this average level of child PTSD symptoms at the family level. An association was found for the aggregated mean of child-reported family functioning. The small overlap between PTSD symptoms of children from the same family may thus be partly explained by their combined perception of the family’s functioning. A supportive family environment, with a good mutual understanding between family members and low levels of conflict, may protect children from the emotional consequences of having a parent with cancer to some extent. However, previous studies (Williamson et al., 2017) as well as the current study suggest only a minor contribution of the family environment. This suggests that child PTSD symptoms are mainly individually experienced and determined by individual appraisals and emotion regulation. In contrast to general family functioning, no significant effect was found for family communication at the individual nor at the family level. This was unexpected, given that many previous studies found poorer family communication to be related to child adjustment problems (Huizinga et al., 2005a, 2011; Lindqvist et al., 2007). As indicated above, this may be the result of controlling for trauma-related cognitions and emotion regulation difficulties on the child level. Also, overall family communication appeared to be good in the families under study, with only limited variation. Finally, the current assessment of family communication showed no optimal reliability for this age group and focused on the general exchange of information between family members (e.g., whether family members are honest with each other) rather than communication about cancer-related experiences. The beneficial effects of informing children openly about their parent’s diagnosis are highlighted in the literature (Ellis et al., 2017; Morris et al., 2016). It would be interesting for future studies to examine cancer-related family communication in relation to child posttraumatic stress.

Strengths of this study include the involvement of both parents and multiple children per family and the simultaneous inclusion of individual and family factors. However, there are also several limitations. First, the use of a convenience sample may have resulted in a sampling bias. For example, participating children were by definition informed about their parent’s cancer diagnosis and willing to share their experiences, which reflects an open family communication. Results may therefore be less applicable to children who are not informed or not willing to participate. On the other hand, parents may also have decided to participate in the study because they were concerned about their children. Second, the sample consisted mainly of families in which the mother had cancer. Although this is common in research on parental cancer (Krattenmacher et al., 2012), this compromises the generalizability of our findings to families in which a father has cancer. Third, generalizability is also limited due to homogeneity of the sample in terms of ethnic origin and parental education. Fourth, a broad child age range was used. Although child age generally does not predict child adjustment (Krattenmacher et al., 2013), the age range crosses several developmental stages. Children may experience their parent’s cancer differently based on their cognitive and social-emotional development. Future studies may examine this more in-depth. Fifth, shared method variance may have inflated some of the associations. In addition, the use of child-reports on family functioning and communication was valuable in capturing the child’s perspective, but parent-based reports may have provided different views. Future research should ideally use assessments from multiple sources (e.g., self-report and interview-based, and child- and parent reported) of the variables of interest. Sixth, although the sample size was relatively large compared with other studies in families confronted with parental cancer, especially findings on the family level should be interpreted with caution, given the sample size. Finally, the cross-sectional nature of the study limits causal inferences. Studies with repeated measurements examining cross-lagged paths between the constructs are needed to determine the direction of effects.

In conclusion, the current study indicates that children from the same family show individual differences in PTSD symptoms when a parent has cancer. Child trauma-related cognitions and emotion regulation difficulties are related to these symptoms. Clinically, this suggests that interventions for children with severe PTSD symptoms requiring psychological support should assess and target both of these factors. Evidence-based therapies, such as trauma-focused cognitive behavioral therapy, are indicated in this regard (Diehle et al., 2015b; Morina et al., 2016). For lower-intensity support, such as psychoeducation to families, integrating information about cognitive restructuring and helpful emotion regulation strategies could also be relevant. In addition, parents may be encouraged and supported to address their children’s appraisal of parental cancer and its consequences and to support them in acknowledging and managing their emotions.

## Acknowledgments

We would like to thank all parents and children who participated in this study. All master-students are appreciated for their contribution to the data collection and we thank Rens van de Schoot for his statistical advice.

## Funding

Financial support for this study was provided by Pink Ribbon (grant 2014-193).

*Conflicts of interest*: None declared.

## Footnotes

1 Regarding the age criterion, some exceptions were made for children slightly below or above the 8–18 years range, when parents and researchers considered their participation to be appropriate (i.e., eight children of 6 or 7 years old were regarded cognitively capable to complete the questionnaires and six children above 18 years still lived at home).
